# Age Differences in Recovery Rate Following an Aerobic-Based Exercise Protocol Inducing Muscle Damage Among Amateur, Male Athletes

**DOI:** 10.3389/fphys.2022.916924

**Published:** 2022-06-13

**Authors:** Irit Markus, Keren Constantini, Nir Goldstein, Roee Amedi, Yael Bornstein, Yael Stolkovsky, Merav Vidal, Shahar Lev-Ari, Roy Balaban, Stav Leibou, Tamar Blumenfeld-Katzir, Noam Ben-Eliezer, David Peled, Yaniv Assaf, Dennis Jensen, Naama Constantini, Gal Dubnov-Raz, Israel Halperin, Yftach Gepner

**Affiliations:** ^1^ Department of Epidemiology and Preventive Medicine, School of Public Health, Sackler Faculty of Medicine, and Sylvan Adams Sports Institute, Tel-Aviv University, Tel-Aviv, Israel; ^2^ Department of Health Promotion, School of Public Health, Sackler Faculty of Medicine, Tel-Aviv University, Tel-Aviv, Israel; ^3^ Department of Clinical Microbiology and Immunology, Sackler School of Medicine, Tel Aviv University, Tel-Aviv, Israel; ^4^ Sackler School of Medicine, Tel Aviv University, Tel-Aviv, Israel; ^5^ Department of Biomedical Engineering, Tel Aviv University, Tel Aviv, Israel; ^6^ Sagol School of Neuroscience, Tel Aviv University, Tel-Aviv, Israel; ^7^ Center for Advanced Imaging Innovation and Research (CAI2R), New-York University Langone Medical Center, New York, NY, United States; ^8^ Department of Neurobiology, Faculty of Life Sciences, Tel-Aviv, Israel; ^9^ The Strauss Center for Neuroimaging, Tel Aviv University, Tel Aviv, Israel; ^10^ Clinical Exercise & Respiratory Physiology Laboratory, Department of Kinesiology and Physical Education, Faculty of Education, McGill University, Montreal, QC, Canada; ^11^ Shaare Zedek Medical center affiliated to the Hebrew University, Jerusalem, Israel; ^12^ Sports and Exercise Medicine Clinic, Sheba Medical Center, Sackler Faculty of Medicine, Tel Aviv, Israel

**Keywords:** aging, downhill running, muscle damage, inflammation, pain perception, recovery

## Abstract

**Purpose:** Compare recovery rates between active young (Y) and middle-aged (MA) males up to 48H post aerobically based, exercise-induced muscle damage (EIMD) protocol. A secondary aim was to explore the relationships between changes in indices associated with EIMD and recovery throughout this timeframe.

**Methods:** Twenty-eight Y (*n* = 14, 26.1 ± 2.9y, 74.5 ± 9.3 kg) and MA (*n* = 14, 43.6 ± 4.1y, 77.3 ± 12.9 kg) physically active males, completed a 60-min downhill running (DHR) on a treadmill at −10% incline and at 65% of maximal heart rate (HR). Biochemical, biomechanical, psychological, force production and muscle integrity (using MRI diffusion tensor imaging) markers were measured at baseline, immediately-post, and up to 48H post DHR.

**Results:** During the DHR, HR was lower (*p* < 0.05) in MA compared to Y, but running pace and distance covered were comparable between groups. No statistical or meaningful differences were observed between groups for any of the outcomes. Yet, Significant (*p* < 0.05) time-effects within each group were observed: markers of muscle damage, cadence and perception of pain increased, while TNF-a, isometric and dynamic force production and stride-length decreased. Creatine-kinase at 24H-post and 48H-post were correlated (*p* < 0.05, *r* range = −0.57 to 0.55) with pain perception, stride-length, and cadence at 24H-post and 48H-post. Significant (*p* < 0.05) correlations were observed between isometric force production at all time-points and IL-6 at 48H-post DHR (*r* range = −0.62 to (−0.74).

**Conclusion:** Y and MA active male amateur athletes recover in a comparable manner following an EIMD downhill protocol. These results indicate that similar recovery strategies can be used by trainees from both age groups following an aerobic-based EIMD protocol.

## 1 Introduction

It is well established that aging has deleterious effects on recovery rate following various exercise protocols (Dedrick and Clarkson, 1990; Ploutz-Snyder et al., 2001). This is mainly because aging is associated with decreased muscle mass and function, increased low-grade chronic inflammation, and weakened immune function (Chung et al., 2009; Woods et al., 2012; Lavin et al., 2020). Moreover, some research suggests that middle-aged individuals are less likely to engage in physical activity compared to younger individuals (Shin et al., 2018). In addition, previous studies showed that lifelong chronic physical activity can reduce chronic inflammation and improve immune and muscle functions (Minuzzi et al., 2019; Lavin et al., 2020). Studies that have compared the recovery rates between younger and middle-aged or older (>50y) trained individuals following resistance exercise protocols, found that the decline in recovery rates begin at ∼40 years of age (Korhonen et al., 2006; Fell and Williams, 2008; Gordon et al., 2017). For example, a comparison in recovery rates between younger and older sedentary adults following an eccentric resistance exercise protocol, found that the older adults suffer from higher rates of eccentric-induced muscle dysfunction (Ploutz-Snyder et al., 2001). To date, however, most such research has focused on resistance exercise. It remains to be determined whether endurance modes of exercise have similar age-related effects on recovery rate as resistance exercise among young and middle-aged individuals.

The mean age of recreational athletes participating in endurance sports increased over the last few decades, emphasizing the importance of developing a deeper understanding of the recovery rates in middle-aged and older individuals (Lehto, 2016). This is, in part, because insufficient recovery following exercise may result in impaired immune function (Peake et al., 2017) and cardiovascular instability (Romero et al., 2017). Subjective measures such as perception of pain and rate of fatigue were reported to be significantly higher in older (45 ± 6y) compared to younger (24 ± 5y) group of well-trained athletes following three consecutive days of 30-min cycling time trials (Fell et al., 2008). However, changes in objective outcomes, including range of motion, markers of muscle damage such as levels of circulating muscle proteins, and performance tests did not differ between younger (27 ± 2) and older (58 ± 2) trained adults following a 45-min downhill run. That study (Hayashi et al., 2019), however, did not assess inflammatory markers or examine changes in muscle integrity (for example via magnetic resonance imaging; MRI) in order to evaluate differences in the effect of exercise-induced muscle damage (EIMD) between age groups at the muscle fibers level.

Unaccustomed, aerobic-based exercise that predominantly includes eccentric contractions can cause structural muscle damage within the activated muscle fibers (Clarkson and Hubal, 2002; Tee et al., 2007). At the cellular level, the consequences of EIMD may include damage to structural proteins, myofibrils and muscle cell membranes, and thus impair excitation-contraction coupling (Fernandes et al., 2019; Markus et al., 2021). Subsequently, muscle strength and function are likely to be impaired (Dedrick and Clarkson, 1990; Lavin et al., 2020). This implication can also affect kinematics indices, as previous studies showed cadence and stride length are considerably altered following EIMD protocols (Kyrolainen et al., 2000; Vernillo et al., 2015). These kinematics changes could therefore shed light on the recovery processes following EIMD induced by running protocols. Taken together, quantifying the aforementioned damaging effects on muscle strength and kinematic indices can provide an effective means to identify age-related differences in recovery rates following muscle-damaging exercise (Pokora et al., 2014; Hayashi et al., 2019). Specifically, the recovery rate following EIMD can be assessed by objective evaluation of muscle proteins and inflammatory markers in the circulation, reduction in muscle strength, biomechanical measures, and performance tests, and by subjective measures such as perception of pain and rating of fatigue. Another objective method to directly assess muscle damage is by diffusion tensor imaging (DTI), which is a highly sensitive and non-invasive MRI technique that can detect exercise-induced changes in muscle structure by quantifying the magnitude and direction of water diffusion and its direction within the muscle fiber (Froeling et al., 2015; Gepner et al., 2017). To date, no studies used DTI to evaluate the effects of age on the rate of recovery following EIMD. Furthermore, to the best of our knowledge, the relationship between changes in the various biochemical, performance, biomechanical, and perceptual markers throughout the recovery period from an aerobic-based protocol causing EIMD remains unclear.

Thus, the primary aim of this study was to compare biochemical, performance, biomechanical and perceptual markers as well as muscle integrity following EIMD between young (Y) and middle-aged (MA) amateur male athletes. We chose these families of variables as they are strongly linked to recovery rates following EIMD protocols (Kyrolainen et al., 2000; Froeling et al., 2015; Gordon et al., 2017; Hayashi et al., 2019; Markus et al., 2021). We hypothesized that the degree of EIMD—as measured by biochemical, biomechanical, perceptual and muscle integrity markers—would be greater in MA amateur athletes compared to their Y counterpart, and that the abovementioned parameters will return to baseline levels earlier in the latter group compared to the former. A secondary aim was to explore the relationships between changes in these indices immediately post and up to 48H following a downhill running protocol designed to induce muscle damage.

## 2 Methods

### 2.1 Study Population

Twenty-eight apparently healthy, recreationally competitive male runners and triathletes were recruited to participate in this study and were divided into two age-based groups: a young group (Y, *n* = 14; age range 18–30 years old), and a middle-aged group (MA, *n* = 14; age range 35–50 years old). All participants performed endurance exercise training at least four times per week and regularly competed in long distance running and/or triathlon events. Exclusion criteria included smoking, prescribed medications, or a self-reported history of chronic pulmonary, cardiac, metabolic, or orthopedic conditions.

### 2.2 Ethical Approval

The nature of the experiments was explained to all participants who provided written informed consent prior to study initiation. The study was approved by Shaare Zedek Medical Center (IRB#0345–19) and Tel Aviv University Ethical committee (#0000400–4), and is registered in www.ClinicalTrial.gov (NCT04025723).

### 2.3 Experimental Design

**FIGURE 1:**
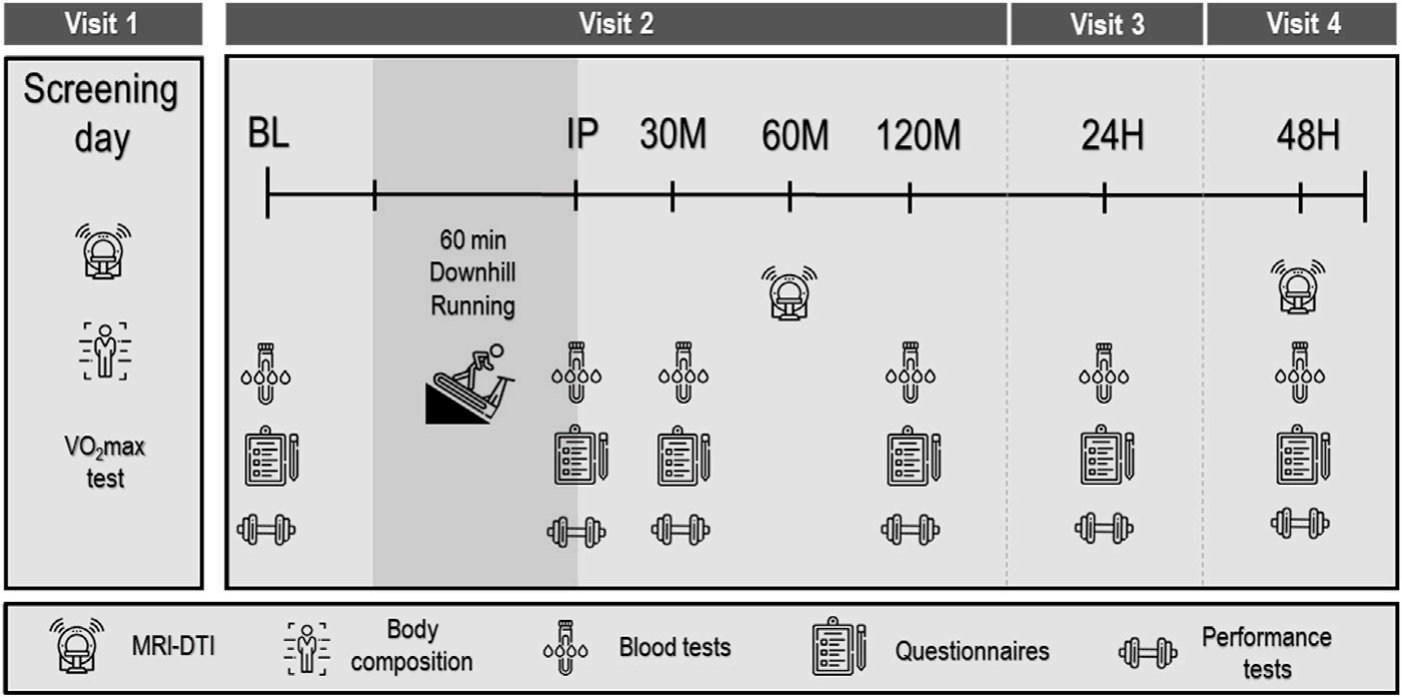
Experimental protocol. Baseline (BL), immediately post (IP), 30 min (30 M), 120 min (120 M), 24 h (24H) and 48 h (48H) post downhill running protocol. VO2max: maximal O2 consumption; MRI-DTI: magnetic resonance imaging diffusion tensor imaging.

Participants visited the laboratory on four separate occasions (Figure 1). Visit 1, which served to screen participants, included filling an inform consent, health and physical activity level questionnaires, baseline MRI scan of the thigh muscles, anthropometric measurement, and a graded running exercise test to volitional exhaustion (i.e., maximal O_2_ consumption [VO_2_max] test). Visits 2–4 were performed on three consecutive days, while Visits 1 and 2 were separated by at least 48 h and all visits were completed within 14 days. During Visit 2 participants initially underwent a series of baseline (BL) measurements that included—in the following order—blood tests, questionnaires (visual analog scale of muscle soreness and rate of fatigue), force production/performance tests (counter movement jump, maximal isometric voluntary contraction), and a biomechanical assessment). Next, participants completed a 60-min downhill running (DHR) protocol, as detailed below. To assess recovery rate, the same series of measurements performed prior to the DGR protocol was conducted immediately (IP), 30 min (30 M-post), 120 min (120 M-post), 24 h (24H-post), and 48 h (48H-post) post the DHR protocol (i.e., a blood draw, assessment of running biomechanics and perceptual questionnaires, force production/performance tests). To assess muscle fiber integrity, MRI-DTI scans were performed on the screening day (prior to the DHR protocol), 60 M-post and 48H-post the DHR protocol (Visits 1, 2 and 4, respectively). Participants were instructed to refrain from consuming alcohol and caffeine, and avoid exercising for 24 h before lab visits 1 and 2 and between visits 2–4. Participants were also asked to fast for two hours prior to each lab visit.

### 2.4 Graded Exercise Test

On visit 1, participants performed a graded exercise test to exhaustion on a motorized treadmill (Saturn 100/300, h/p/cosmos, Nussdorf-Traunstein, Germany) using an individualized protocol. For the first 5–6 min of the test, speed was increased every 1 min until reaching a comfortable speed (i.e., a speed at which each participant could easily run for 1 h). Then, the grade of the treadmill was increased 2% every 1 min until participants reached volitional exhaustion, defined as the point during exercise at which the participant (voluntarily) expressed inability to continue exercising despite strong verbal encouragement. Ventilatory and metabolic measurements were collected during the graded protocol using breath-by-breath analysis (Quark Cardiopulmonary Exercise Testing, Cosmed, Rome, Italy) while subjects breathed through an oro-nasal facemask (7450 Series, Hans Rudolph, Kansas City, MO, United States). Heart rate (HR) was continuously monitored using a chest strap (Garmin®, model Acc, HRM-Dual, Kansas City, MO, United States). VO_2_max was determined as the highest 30-s average O_2_ uptake (VO_2_) achieved during exercise (as previously done by other, such as Bailey et al. (2009)), while meeting two of the following three criteria: 1) an HR ≥ 90% age-predicted maximum, 2) an RER ≥1.10, and 3) a plateau in VO_2_ ≤ 150 ml with increased workload (Howley et al., 1995). Maximal HR (HRmax) was determined as the highest HR recorded during the test.

### 2.5 Downhill Running Protocol

Participants performed a 5-min warm-up at 0% grade while speed was increased every 30 s until the target HR of 65% of HRmax was reached. Following the warm-up, the slope of the treadmill was reduced to −10% (Byrnes et al., 1985; Peake et al., 2005; Hayashi et al., 2019) for 55 min with the running speed adjusted throughout the test so that each participant’s HR remained at 65% HRmax (±5 beats/min). Every 10 min, participants were asked to rate their perceived exertion (RPE) on the Borg 6–20 scale (Borg, 1982). HR, speed (km/h), and distance (km) were recorded every 10 min.

### 2.6 Anthropometric Assessment

Body mass, waist circumference, fat mass and lean body mass were measured using bioelectrical impendence analysis (BIA; SECA^©^ Medical Body Composition Analyzer, mBCA 515, Hamburg, Germany). Height was measured using a stadiometer (SECA 274).

### 2.7 Force Production/Performance Tests

The participants were familiarized with the performance tests on Visit 1 and, following a standardized warm-up (50% effort for two times), were asked to complete these tests at all six time points.

#### 2.7.1 Maximal Voluntary Isometric Contraction (MVC)

Participants performed three knee extensions MVCs with one minute rest between each of the three trials. Lying on a treatment bed in the supine position, a padded strap was secured around their right ankle with the other side of the strap secured to a metal rod in the bed with the knee fixed at a 90° angle. Participants were instructed to extend their knee as forcefully as possible for 5 s. Force data were collected by the strain gauge (Chronojump®, Barcelona, Spain) and recorded by Chronojump® software at a sampling frequency of 80 Hz. The highest value obtained during the three trials was used. If the two best measurements differed by more than 10%, participants were asked to perform a fourth trial.

#### 2.7.2 Countermovement Jump (CJM)

Participants were instructed to put their hands on their waist and jump as high as possible, while maintaining their legs shoulder-width apart, with no restrictions on how low they needed to squat prior to jumping. This test was repeated three times with one minute of rest between jumps. Jump height was calculated by flight time, using a jumping gate (Optogait, Microgate, Bolzano, Italy). The highest value obtained during the three trials was used. If the two best measurements differed by more than 10%, participants were asked to perform a fourth trial.

### 2.8 Biomechanics Assessment

At all six time points (BL, IP, 30 M-post, 120 M-post, 24H-post and 48H-post) participants from both age groups were asked to walk for 1 min at a fixed speed of 6 km/h and run for 2 min at 9 km/h at grade of 0% on a treadmill (Gaitway^©^ mad, h/p/cosmos, Nussdorf-Traunstein, Germany). Stride length and cadence were measured using built-in pressure sensors within the treadmill (Zebris©, Munich, Germany). Data were recorded during the last 30 s of the running phase using Noraxon software (Noraxon Inc., Scottsdale, AZ, United States) at a frequency of 100 Hz and analyzed using Myoforce (Noraxon Inc., Scottsdale, AZ, United States).

### 2.9 Perceptual Questionnaires

#### 2.9.1 Perception of Pain in the Quadriceps Muscle

Prior to all performance and biomechanics tests, participants were asked to quantify their degree of pain in the quadriceps muscle of the right thigh on a 15 cm v*isual analog scale* (VAS). Participants provided their levels of pain by making a mark on a horizontal line that ranged from “no pain at all” to “maximal pain” anchored at each end of the VAS.

#### 2.9.2 Rate of Fatigue (ROF) Scale

Prior to all performance and biomechanics tests and following the VAS questionnaire, participants were asked to rate their level of fatigue, which was measured on an 11-point numerical scale with empirically derived accompanying descriptor and diagrammatic components of ROF (Micklewright et al., 2017).

#### 2.9.3 Rating of Perceived Exertion (RPE)

Every 10 min during the DHR protocol, participants were asked to rate their perceived exertion on a numerical scale from 6–20 by the question “*Rate the level of effort you are currently feeling*”, where 6 represents “no exertion at all” and 20 is “maximal exertion” (Borg, 1982).

### 2.10 Blood Collection and Biochemical Analysis

A total of 20 ml of blood was obtained at each of the six time points in two, 10 ml Vacutainer® tubes: one containing ethylenediamine tetraacetic acid (EDTA) and the other a serum tube, for plasma and serum samples, respectively. Plasma samples were subsequently centrifuged twice at 1,500 g for 10 min at 4°C, while serum was kept at room temperature for 1 h then centrifuged at 1,300 g for 10 min. Samples were aliquoted into 1.8-ml microcentrifuge tubes and frozen at −80°C until analyzed. Interleukin (IL) 6, IL-10, tumor necrosis factor (TNF-α), and IL-1 receptor antagonist (RA) were analyzed by a high-sensitivity cytokine multiplex assay (Luminex, Cat no. FCSTM09-02; R & D Systems, Inc., Minneapolis, MN, United States) on a MAGPIX instrument (Luminex, Austin, TX, United States) according to the manufacturer’s instructions. Extrapolation analysis for missing data was performed by a linear model in Analyst v5.1 Flex software (MILLIPLEX™, Carlisle, MA). Creatine kinase (CK), lactic dehydrogenase (LDH), and C-reactive protein (CRP) were analyzed by a Roche clinical chemistry and immunochemistry analyzer (Roche Diagnostics, Cobas® c111, Indianapolis, IN, United States).

### 2.11 MRI-Scans

DTI scans were performed on Magnetom Prisma 3T Siemens scanner at the Alfredo Federico Strauss Center located at Tel Aviv University. MRI data were obtained at BL, 60 M-post and 48H-post the DHR protocol. The distal quarter of the right thigh was positioned at the center of the coil and was scanned for 45 min. Four muscles were chosen to evaluate the anterior (rectus femoris and vastus lateralis) and posterior (biceps femoris and semitendinosus) compartments of the thigh muscles. Muscle integrity was assessed using DTI, a protocol that is sensitive to subclinical signs of muscle injury and changes in the tissue, as we and other have previously shown (Froeling et al., 2015; Oudeman et al., 2016; Biglands et al., 2020). Data obtained from MRI DTI scans can be used to calculate parameters that are sensitive to tissue changes following EIMD (Froeling et al., 2015), such as fractional anisotropy and mean diffusivity by evaluating the quality and direction of water diffusion in a given voxel, thus providing information about the integrity of the area of skeletal muscle scanned (Keller et al., 2020).

### 2.12 Statistical Analysis

Differences in baseline characteristics between study groups were compared using *t*-test for continuous variables, or chi-square tests for categorical variables. For each marker of muscle damage (e.g., biochemical, force production/performance, rating of fatigue and perception of pain, biomechanical, and MRI-DTI parameters), changes at each time point following the DHR protocol relative to baseline (∆%) were analyzed using a two factor (time x group) repeated measures analysis of variance (ANOVA). In the event of a significant F-ratio, least significant difference (LSD) *post-hoc* analyses were used for pairwise comparisons. Correlations between selected muscle damage and inflammatory biomarkers (e.g., CK, CRP, IL-6, TNF-a), performance (CMJ, MVC) and subjective (VAS) measures were evaluated using Pearson *r* correlations. These correlations were performed on data from the entire cohort combined and, except for VAS, assessed as percentage change relative to baseline (∆%). Sample size calculation was based on changes in muscle strength as a result of EIMD between young and middle-aged males (Gordon et al., 2017) using a Winpepi software. On the basis of *α* = 0.05 and *β* = 0.2 with 80% power, the minimum number of subjects that were needed was 14 in each age group, with a total of 28 participants. Significance required an alpha level of *p* ≤ 0.05. All data were analyzed by using IBM SPSS Statistics v25 software (SPSS, Inc., Chicago, IL, United States). Data are reported as mean ± SD, unless otherwise specified.

## 3 Results

**FIGURE 2:**
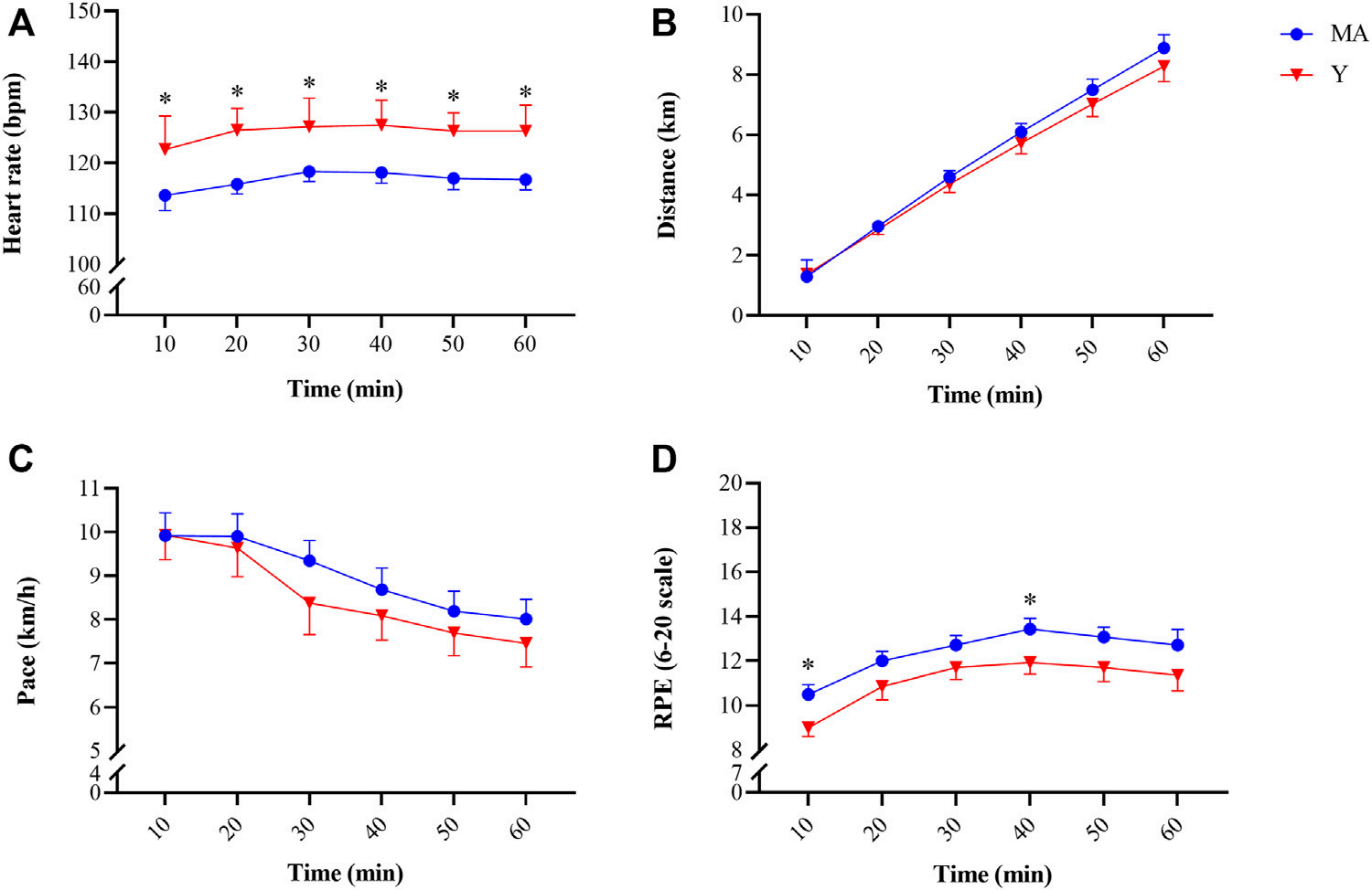
Changes in heart rate [panel **(A)**], distance covered [panel **(B)**], pace [panel **(C)**] and rating of perceived exertion [panel **(D)**] recoded every 10 min during the 60-min downhill running protocol. * Significant (*p* < 0.05) difference between young (Y; red triangle) and middle-age (MA; blue circles) groups. All data are reported as mean ± SEM.

Study population characteristics, including training experience, weekly training volume are presented in Table 1. As expected, HRmax was significantly (*p* < 0.001) higher in the Y group compared to the MA group. Accordingly, during the DHR protocol HR was consistently higher in the Y group vs. MA (*p* < 0.001; Figure 2). RPE was only significantly (*p* < 0.05) higher in the MA group compared to Y at the 10 and 40 min marks (Figure 2) while distance and pace were not different between age groups.

**TABLE 1:** Baseline characteristics of the study population across age groups

	Young (18–30y)	Middle age (35–50y)	*p* value
	*n* = 14	*n* = 14	
Age (y)	26.1 ± 2.9	43.6 ± 4.1	< 0.001
Height (cm)	174.5 ± 0.1	175.5 ± 0.1	0.632
Weight (kg)	74.5 ± 9.3	77.3 ± 12.9	0.520
BMI (kg/m^2^)	24.1 ± 2.5	25.3 ± 3.7	0.311
Waist circumference (cm)	82.8 ± 6.2	91.4 ± 10.4	0.014
Fat mass (%)	14.1 ± 4.3	19.2 ± 9.0	0.083
Lean body mass (kg)	60.4 ± 7.1	58.8 ± 7.1	0.552
Resting heart rate (bpm)	56 ± 9	52 ± 10	0.337
Maximal heart rate (bpm)	189 ± 7	174 ± 9	<0.001
VO_2_max (ml/kg/min)	50.4 ± 6.1	46.5 ± 6.4	0.111
Systolic blood pressure (mmHg)	117 ± 11	122 ± 8	0.159
Diastolic blood pressure (mmHg)	69 ± 6	75 ± 8	0.053
Night time sleep (h)	6.8 ± 0.9	6.1 ± 1.2	0.087
Training years (y)	4.6 ± 4.3	10.2 ± 8.5	0.039
Weekly exercise (min)	558 ± 268	442 ± 183	0.206
Reported weekly running (km)	40 ± 27	54 ± 27	0.215
Resistance training (Yes)	9 (60.0%)	6 (40.0%)	0.363
Downhill experience (Yes)	0 (0.0%)	11 (71.4%)	<0.001

All data are reported as mean ± SD. Night time sleep—average of three nights.

### 3.1 Performance Tests: MVC and CMJ

**FIGURE 3:**
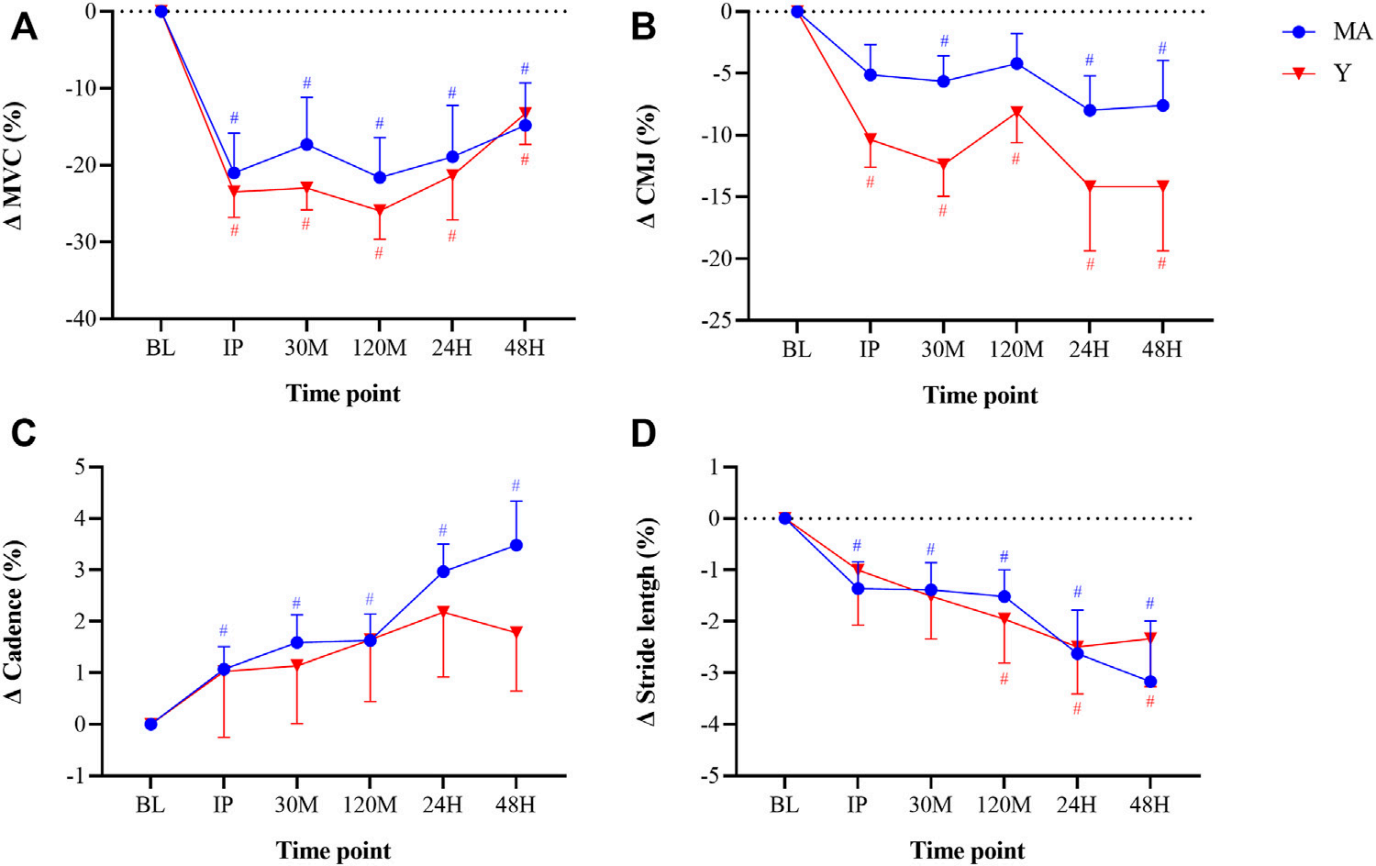
Changes in maximal voluntary contraction [MVC; panel **(A)**], counter movement jump [CMJ; panel **(B)**], cadence [panel **(C)**], and stride length [panel **(D)**] relative to baseline (BL). Cadence and stride length were recorded while running at a constant pace of 9 km/h. Measurements were taken as BL, immediately post (IP), 30min (30 M), 120 min (120 M), 24 h (24H) and 48 h (48H) following a 60 min downhill running protocol. Young (Y; red triangle) and middle-age (MA; blue circles) groups. Dotted horizontal line represents no change compared to BL. # Significant (*p* < 0.05) difference compared with BL within each group. All data are reported as mean ± SEM.

No group effect was found for either MVC or CMJ. A significant time effect (*p* < 0.001) was observed for peak torque during MVC in both the MA and Y groups (F = 6.274 and F = 12.356, respectively; Figure 3). A significant (*p* < 0.05) time effect was also observed in CMJ for both the MA and Y groups (F = 3.961 and F = 4.125, respectively). The Y group showed a significant (*p* < 0.05) reduction in peak power of ∼10% at all time points post, while MA showed a moderate yet significant (*p* < 0.05) decrease of ∼5% only at 30 M-post, 24H-post and 48H-post.

### 3.2 Biomechanics Assessment: Cadence and Step Length

No group effect was found for either step length or cadence. Significant (*p* < 0.05) time effects were detected in both MA and Y groups for step length (F = 6.419 and F = 2.932, respectively; Figure 3). A significant (*p* < 0.05) time effect for cadence was observed only in the MA group (F = 7.393).

### 3.3 Perceptual Questionnaires: Rating of Fatigue and Soreness

No group effect was found for either step length or cadence. A significant (*p* < 0.05) time effect (F = 2.752) was observed for the ROF questioner in the Y group and was significantly higher, at 30 M-post and 120 M-post compere to baseline. A significant (*p* < 0.05) time effect for the VAS questioner, was observed in both groups (MA: F = 11.236; Y: F = 18.466) and was significantly higher at IP, 30M, 120M, 24H and 48H-post protocol in both groups.

### 3.4 Biochemical Analysis: Blood Markers of EIMD

**FIGURE 4:**
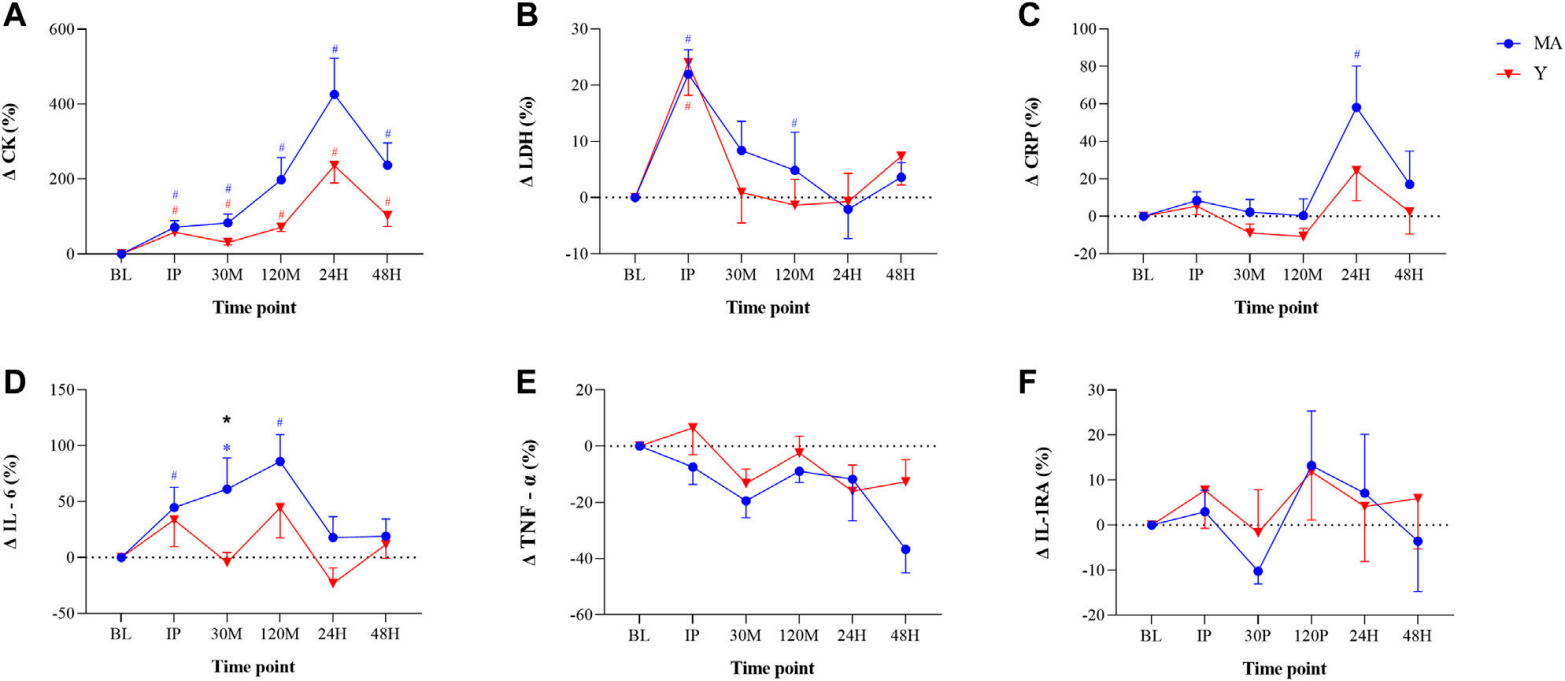
Changes in blood markers of muscle damage and inflammation: Creatine kinase [CK; panel **(A)**], Lactate dehydrogenase [LDH; panel **(B)**], C-reactive protein [CRP; panel **(C)**] Interleukin-6 [IL-6; panel **(D)**], Tumor necrosis factor-α [TNF-α; panel **(E)**], and IL-1 receptor antagonist [IL-1RA; panel **(F)**] relative to baseline (BL). Measurements were taken as BL, immediately post (IP), 30 min (30 M), 120 min (120 M), 24 h (24H) and 48 h (48H) following a 60 min downhill running protocol. Young (Y; red triangle) and middle-age (MA; blue circles) groups. Dotted horizontal line represents no change compared to BL. # Significant (*p* < 0.05) difference compared with BL within each group. * Significant (*p* < 0.05) difference between groups at given time point. All data are reported as mean ± SEM.

No group effect was found for blood markers of EIMD. There was a significant (*p* < 0.05) time effect for CK (MA: F = 16.017; Y: F = 16.233) and LDH (MA: F = 4.505; Y: F = 3.813) (Figure 4). CK reached a peak 24H-post DHR protocol, with a more pronounced elevation in the Y group. CRP reached a peak 24H-post DHR protocol, with a more significant elevation only occurring in the MA group.

### 3.5 Inflammatory Markers

There were no differences between age groups in any blood markers at baseline and no group effect was found for inflammatory markers, except for IL-6 at a single time point (see below). A significant (*p* < 0.05) time effect for CRP was observed within MA group (F = 4.067; Figure 4). In both groups, CRP peaked at 24H-post DHR, but the elevation was significant (*p* < 0.05) only for the MA group. In the MA group there was a significant (*p* < 0.05) time effect (F = 4.004) for IL-6. There was a significant (*p* < 0.05) difference in IL-6 between groups at 30 M-post. No time effect was observed for TNF-α and IL-1RA.

### 3.6 MRI-DTI Scans

**FIGURE 5:**
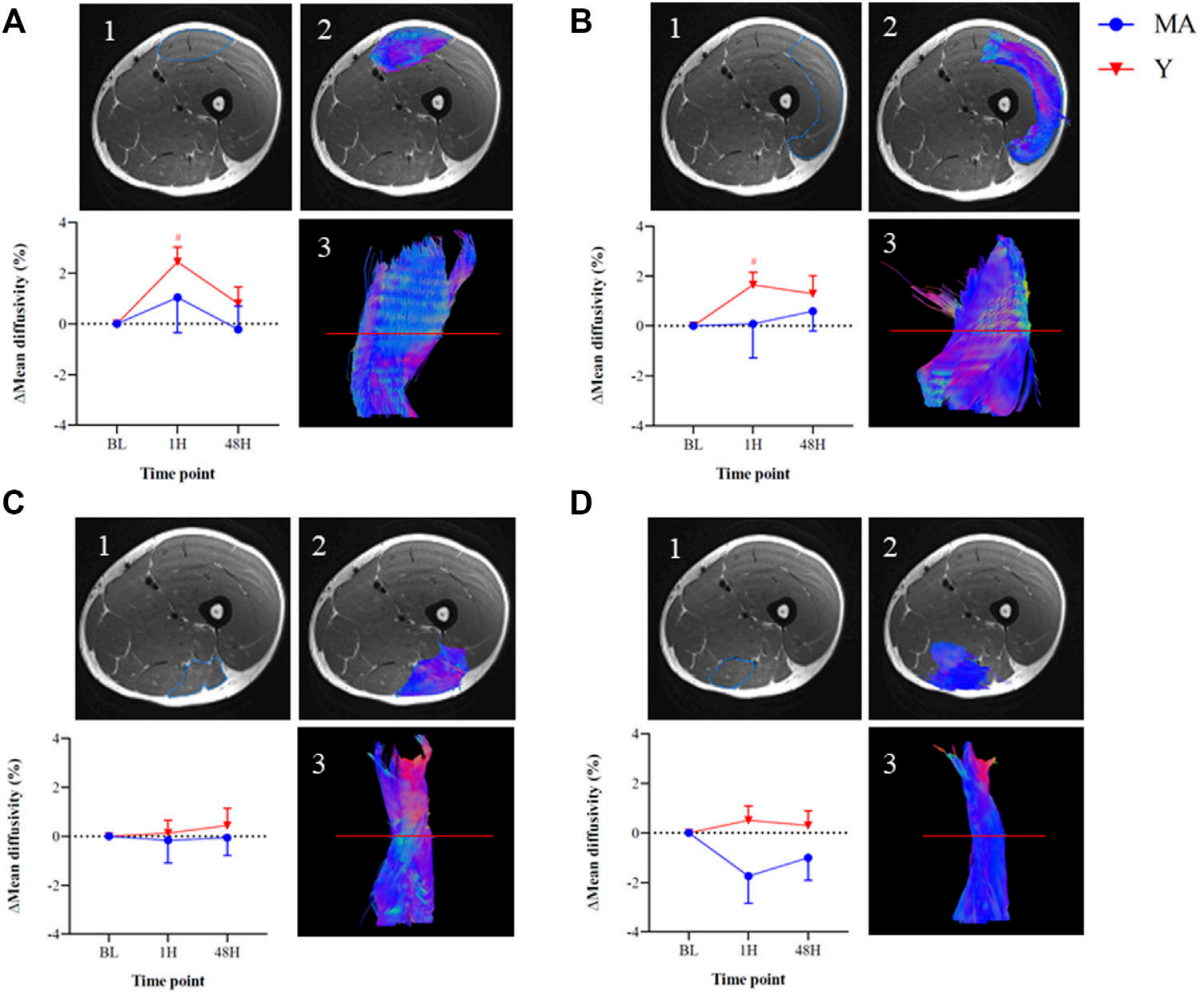
Changes in mean diffusivity within the thigh muscles of the right leg relative to baseline (BL) measured using magnetic resonance imaging diffusion tensor imaging (MRI DTI) imaging: Rectus femoris [panel **(A)**], Vastus lateralis [panel **(B)**], Biceps femoris [panel **(C)**], and Semitendinosus [panel **(D)**]. The images represent an MRI-DTI analysis of the thigh muscles of one representative young participant at BL. 1. Region-of-interest on an axial slice 2. Colored region-of-interest on an axial slice 3. Tractography of individual muscle using DTI analysis on sagittal slice, based on the axial slice (red line). The colors within the images represent; Blue—Superior/cranial or Inferior/caudal, Green—Anterior or Posterior, Red—Distal or Proximal. Measurements were taken as BL, 1 h (1H) and 48 h (48H) following a 60 min downhill running protocol. Young (Y; red triangle) and middle-age (MA; blue circles) groups. Dotted horizontal line represents no change compared to BL. # Significant (*p* < 0.05) difference compared with BL within each group. All data are reported as mean ± SEM.

There was no time or group effect for any of the other MRI analyses (*p* > 0.05). There was a significant (*p* < 0.05) time effect for mean diffusivity in the rectus femoris (F = 7.025) and vastus lateralis (F = 4.515) muscles of the Y group. Changes in the parameters obtained from MRI-DTI analyses are shown in (Figure 5).

### 3.7 Correlations

**FIGURE 6:**
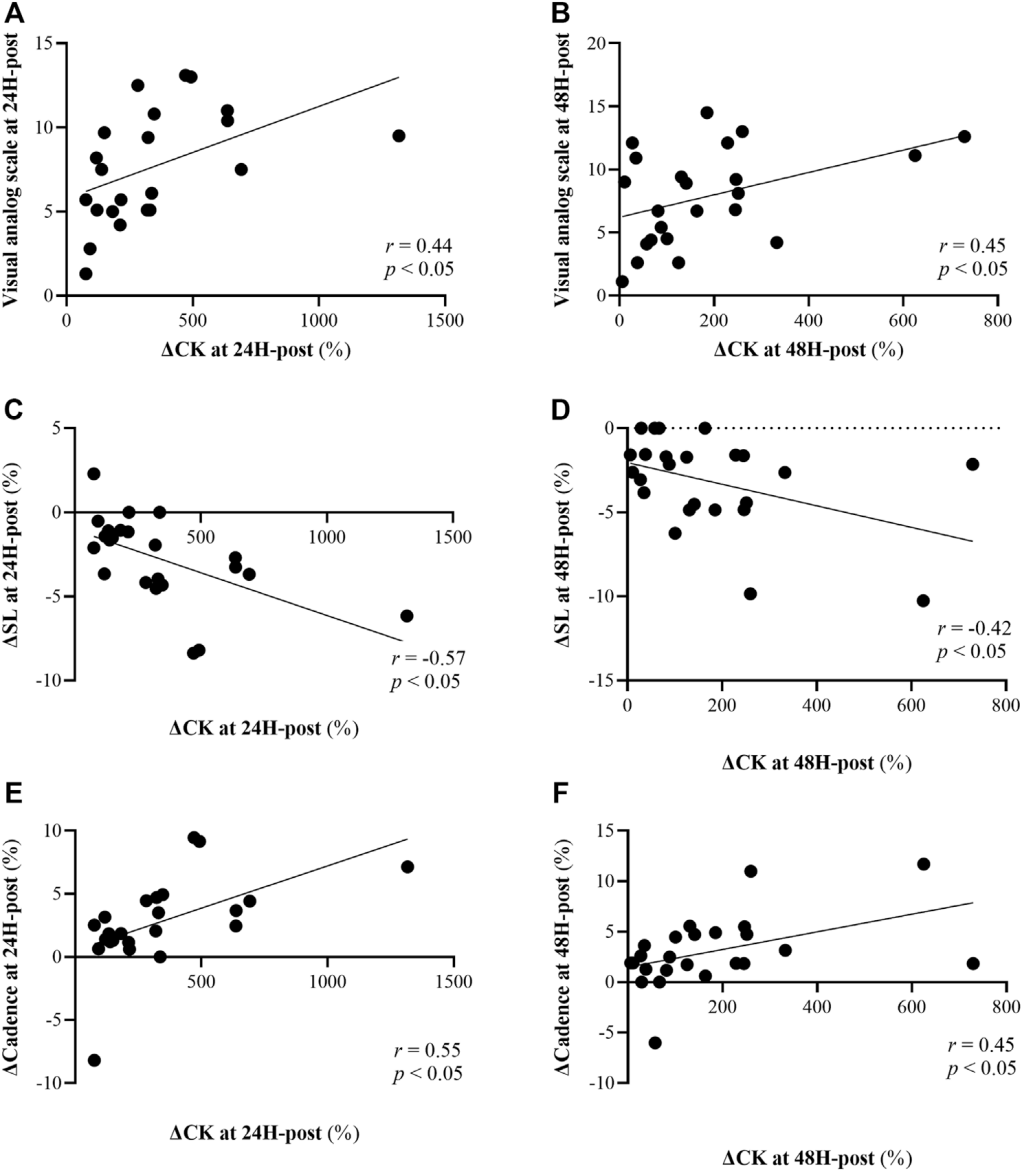
Correlations between changes in creatine kinase (CK) at 24H-post (left panels) and 48H-post (right panels) 60 min downhill running (DHR) protocol relative to baseline and: Visual analog scale (15 point scale) at 24H-post [panel **(A)**] and 48H-post [panel **(B)**] DHR, changes in stride length (SL) at 24H-post [panel **(C)**] and 48H-post [panel **(D)**] DHR compared to baseline, and changes cadence at 24H-post [panel **(E)**] and 48H-post [panel **(F)**] DHR compared to baseline.

Elevated CK concentrations at 24H-post (∆335%) and 48H-post (∆175%) were statistically correlated (*p* < 0.05) with the following measures at 24H-post and 48H-post (Figure 6): increases in VAS (*r* = 0.44 and 0.45, respectively), decreases in stride-length (*r* = -0.57 and -0.42, respectively), and increases in cadence (*r* = 0.55 and 0.45, respectively).

Ratings of perception of pain (assessed via a VAS), were statistically correlated with cadence at 120M, 24H and 48H-post (*r* = 0.45, *r* = 0.43 and *r* = 0.40, respectively; *p* < 0.05 for all). Stride length was negatively correlated with VAS at 120M and 24H-post (*p* < 0.05; *r* = -0.44, *r* = -0.47, respectively).

**FIGURE 7:**
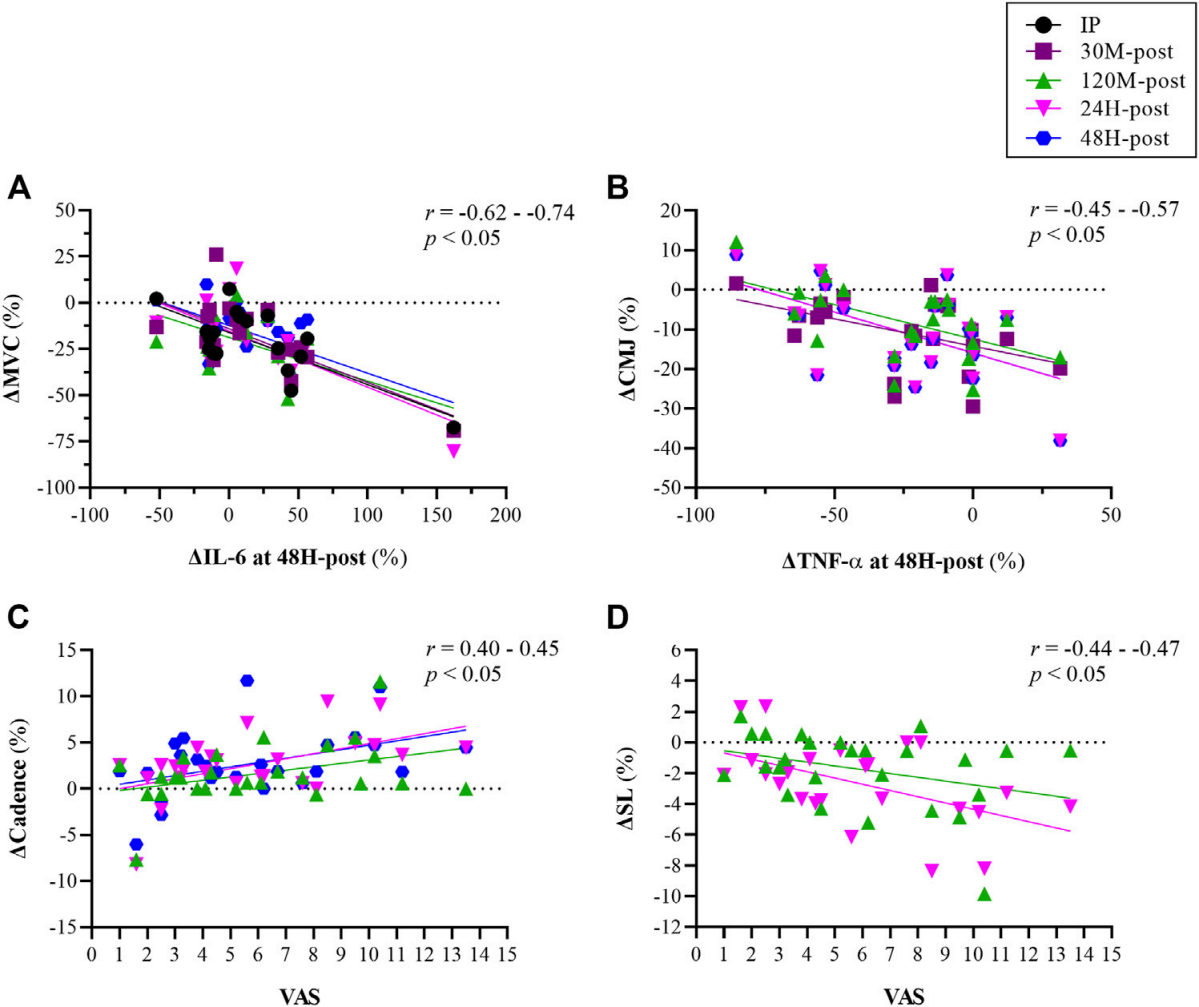
Correlations between changes in inflammatory/biochemical markers [interleukin (IL)-6, Tumor necrosis factor-α (TNF-α)] and changes force production at various time points relative to baseline [panels **(A,B)**], and between subjective perception of pain measured assessed using a visual analog scale (VAS; 15-point scale) and changes in biomechanical indices [cadence panel **(C)**; stride length (SL) panel **(D)**] at various time points relative to baseline. Measurements were taken as baseline, immediately post (IP), 30 min (30 M), 120 min (120 M), 24 h (24H) and 48 h (48H) following a 60 min downhill running protocol. Each color represents corrolation between two variables at given time point (see legend for details).

Significant correlations were also observed between performance measures and inflammatory cytokines: first, elevated levels (∆16%) of IL-6 at 48H-post DHR protocol were statistically (*p* < 0.05) correlated with greater reductions (∆ -14 to -24%) in isometric force production (i.e., MVC) at all time points (*r* = -0.62 to -0.74; Figure 7). Second, changes in the pro-inflammatory cytokine TNF-a at 48H-post were statistically (*p* < 0.05) correlated with changes in dynamic force production (i.e., CMJ) at 30M to 48H-post (*r* = −0.45 to −0.57), where those whose TNF-a levels were reduced to a greater extent were able to better maintain peak CMJ force (Figure 7). No significant correlations were found between MRI-DTI to blood markers, subjective perception or performance.

## 4 Discussion

This study was designed to comprehensively evaluate the differences in recovery rates between Y and MA amateur, male athletes, following an endurance-based protocol intended to induce muscle damage. While all participants demonstrated signs of considerable muscle damage as a result of the DHR protocol, surprisingly, and as opposed to our hypothesis, no differences in any of the outcomes were observed between the Y and MA groups at any given time point following DHR. These similarities in recovery patterns could suggested that an active lifestyle and consistent training throughout many years - as practiced by the MA group in this study - can negate some of the age-related effects associated with slower recovery rates with aging. Additionally, our findings that biomarkers of muscle damage and inflammation were strongly correlated with heightened perception of pain, alterations in running biomechanics and impairments in force production abilities, have important practical implications. Specifically, it can be suggested that subjective scales and performance tests may be carefully used to assess the severity of muscle damage and/or predisposition to altered biomechanical patterns when invasive or advanced measures are unavailable.

Aging is associated with a state of low-grade chronic inflammation and weakened immune function (Chung et al., 2009; Woods et al., 2012; Lavin et al., 2020); thus, the ability to cope with the “physiological load” after EIMD is likely to decline with age. Yet, in our study, no significant differences between the groups were found either at baseline or following the DHR protocol in inflammatory cytokines (e.g., IL-6, IL1-RA, CRP, TNF-a) or markers that are indicative of structural damage (CK and LDH) (Markus et al., 2021) (Figure 4). This finding is consistent with Hayashi et al. (2019), who also reported no differences in CK between young and older trained individuals, despite a peak 24H-post DHR protocol. Additionally, although acute alterations in performance (measured by MVC and CMJ) and running biomechanics (stride length and cadence) were observed following the DHR protocol, values were not different between the groups and followed similar patterns (Figure 3). These findings could suggest that consistent, long-term training, as experienced by the MA group, may potentially blunt age-related effects associated with the severity of and recovery from muscle damage following aerobic-based exercise.

To the best of our knowledge, this is the first study to assess differences in muscle damage between age groups following an endurance protocol via MRI-DTI technique. A scant number of studies have previously used this technique, which serves as a direct assessment of muscle integrity (as opposed, for example, to blood markers and pain scales), to evaluate muscle damage (Cermak et al., 2012; Froeling et al., 2015; Maeo et al., 2017). However, none of these studies compared age-related differences in EIMD or recovery rate. Our results showed a significant increase in mean diffusivity - a marker of microstructure changes - of the anterior department of the thigh muscles (rectus femoris and vastus lateralis) in the Y group following the DHR protocol (Figure 5). These results are similar to previous studies that used MRI to evaluate muscle damage (Froeling et al., 2015; Keller et al., 2020). For instance, Maeo et al. (2017) showed that 45 min of downhill running caused significant muscle damage to the knee extensors and Froeling et al. (2015) demonstrated similar changes in mean diffusivity after a marathon (42.2 km) run. Despite significant alterations in blood markers of muscle damage, muscles of the posterior department of the thigh (biceps femoris and semitendinosus) did not show any changes following the DHR protocol implemented in this study, which contrasts a previous study that examined EIMD following marathon running (Froeling et al., 2015). Future studies should implement the MRI-DTI technique as it is a powerful tool that can give deep insight into the muscle status at the fiber level.

We found a significant correlation between ∆CK and VAS at 24H and 48H-post DHR protocol (Figure 6). Greater ratings of subjective soreness 24–48H post EIMD has been previously shown (Gordon et al., 2017; Hayashi et al., 2019), and are likely related to severe muscle damage and associated elevated levels of CK in the bloodstream. At 24H-post DHR protocol, VAS and ∆CK were each also correlated with biomechanical measurements (Figures 6,7). The observed relationship between CK, a marker of muscle damage, and alterations in running biomechanics is physiologically sound: the micro-tears cause by EIMD, which also result in elevated CK, lead to impairments in muscle function and thus stepping patterns such as stride length and cadence. These findings have important practical implications: as measuring blood markers or gait patterns often may not be a feasible approach, coaches and athletes could instead utilize VAS to assess the severity of muscle damage and/or predisposition to altered biomechanical patterns. Thus, more informed decisions as to whether the athlete is ready to perform another high-intensity workout can be made. It is important to consider the repeated bout effect which is known to attenuate muscle damage markers such as CK (Doma et al., 2017) due to the skeletal, neural and structural protective adaptation (Hyldahl et al., 2017), and therefore this approach should be used with caution.

Higher levels of IL-6 were found to correlate with poorer isometric force production measured via MVC, and depressed TNF-α levels were correlated with better (i.e., less reductions) in dynamic force production measured via CMJ (Figure 7). It could be assumed that since IL-6 is an anti-inflammatory agent, elevated levels of this cytokine could lead to greater reduction in isometric force production. On the other hand, greater reduction in the pro-inflammatory cytokine TNF-α may have resulted in better ability to maintain force during a dynamic, explosive movement such as CMJ. These results emphasize the importance of evaluating recovery following EIMD by investigating the relationships between diverse methods as suggested in previous studies (Bartolomei et al., 2017; Hayashi et al., 2019; Markus et al., 2021).

Our study is unique in its comprehensive and sensitive approach for evaluating the rate of recovery following aerobic-based protocol to induce muscle damage. We trust that the chosen protocol did indeed lead to EIMD due to the significant changes from baseline within each group in nearly all of the markers that have been assessed. While several other studies implemented a DHR protocol as well (Pokora et al., 2014; Hayashi et al., 2019), existing literature mostly consists of resistance-based training protocols (Arroyo et al., 2017; Gordon et al., 2017; Fernandes et al., 2019). This difference in study design could possibly explain the discrepancies between our study and others (Clarkson and Dedrick, 1988; Ploutz-Snyder et al., 2001), as resistance training is expected to load the neuromuscular system such that greater muscular damage occurs compared to aerobic exercise (Markus et al., 2021). Here, we specifically chose an exercise protocol that has been shown to lead to EIMD (Hamada et al., 2005; Peake et al., 2005; Pokora et al., 2014) and is aerobic in nature, as this type of training (i.e., aerobic/endurance) is commonly practiced by recreational and amateur athletes.

A limitation of our study is that we did not include a group of untrained, middle-aged participants, which may have deepened our understanding of the effects of previous training experience on recovery rate between Y and MA individuals. To the best of our knowledge, there are a limited number of studies that examined the effects of and recovery from aerobic-based EIMD between trained to untrained populations. One study Smith et al. (2007) that examined the effects of a 60 min downhill running protocol among untrained young individuals (18–30 y) showed CK values at 24H-post protocol that were nearly double those that we observed at the same time point in the young, trained group (897 ± 97 vs. 476 ± 200 IU/L, respectively). Another comparison between our study to (Smith et al., 2007) showed a threefold increase in IL-6 among the untrained group, IL-6 increased 6 h post protocol to ∼29 pg/ml while in our study, we observed at 120 M-post absolute values of ∼9 pg/ml. While Hayashi et al. (2019) did not report values for blood markers in the young, untrained group included in their study, the reductions in isometric strength following a downhill running protocol experienced by that group are of a smaller magnitude than those observed in our study for trained amateur athletes (10–15% vs. 20–30%, respectively). These results emphasize the attenuated response among trained populations following aerobic-based EIMD. Additionally, we followed the recovery phase only up to 48H post DHR. This decision was partly based on our understanding that participants in our study represent a population that does not usually rest for more than 48H between training sessions. Lastly, since we did not include females in this study, our findings can only be applied to males. Future studies should investigate the age-dependent differences in the extent of muscle damage and recovery rate following an aerobic/endurance-based exercise protocol among females as well.

## 5 Conclusion

Our findings demonstrate that the extent of recovery rate following EIMD, as measured using wide range of parameters, was similar between MA and Y amateur male athletes. While these results do not match our hypothesis, they could indicate that a highly active lifestyle for many years, as practiced by the MA group on this study, might preserve the capacity to recover in a similar manner to younger individuals following muscle damage induced by aerobic exercise. From a practical standpoint, similar approaches may be implemented in reference to recovery from EIMD for both middle-aged and young active males. Our results also indicated strong correlations between biochemical perturbations, heightened perception of muscle pain, and alternation in running biomechanics, and reductions in force production in the 48H following an aerobic-based muscle damaging protocol. Since invasive biochemical and inflammatory/muscle damage blood measures are often unavailable, subjective pain perception scales and force-production tests which can be performed in field settings can be carefully used to assess the severity of muscle damage.

## Data Availability

The original contributions presented in the study are included in the article/Supplementary Materials, further inquiries can be directed to the corresponding author.

## Abbreviations

CK, creatine kinase; CRP, c-reactive protein; EIMD, exercise induced muscle damage; IL1-RA, interleukin receptor antagonist 1; IL-6, interleukin 6; DHR, downhill running; DTI, diffusion tensor imaging; LDH, lactate dehydrogenase; TNF—α, tumor necrosis factor α; VAS, visual analog scale; CMJ, countermovement jump; VO2max, maximal oxygen uptake; MA, middle age; MVC, maximal voluntary contraction; MRI, magnetic resonance imaging; ROF, rate of fatigue; RPE, rated perceived exertion; Y, young.
